# Supplementary material for: The Effects of Peer Competition-Induced Anxiety on Massive Open Online Course Learning: The Mediating Role of the Behavioral Inhibition System
Source: Behav Sci (Basel). 2024 Apr 14;14(4):324. doi: 10.3390/bs14040324 (PMC11047602; doi:10.3390/bs14040324)
Supplement: Supplementary file 1 [file behavsci-14-00324-s001.zip › behavsci-2901234-supplementary.pdf]

Effects of peer competition-induced anxiety on MOOC learning: the mediating role of Behavioral Inhibition System

Supplemental Materials

1) Analysis based on the clinical cut-off points for trait and state anxiety

The clinical cut-off points for TA for males and females are 56 and 57; the clinical cut-off points for SA for males and females are 53 and 55. The results are shown below and are consistent with previous analyses:

In study 1: For TA, we found that 8 participants had TA above the cut-off points, and further analyses revealed: 1) results of analyses within the cut-off point, consistent with previous analyses. Specifically, the differences in TA and SCL between the competition and control groups were not significant, the test scores were better in the competition group (Table S1), and the mediating effect of BIS in TA on SCL on quizzes was significant (β = 0.0268, 95% CI = [0.01, 0.06]). 2) Analyses of data outside the cut-off point show that none of the differences in TA, SCL and test scores between the two groups, no mediate the effect, which may be since the sample is so small.

Table S1 Results of cut-off points analysis of TA in Study 1

	within the cut-off point (N = 101)			without the cut-off point (N = 8)		
	t	df	p	t	df	p
TA	1.399	91	0.165	1.571	6	0.177
SCL	0.692	91	0.491	1.027	6	0.352
Test scores	-2.561	91	0.012	-0.180	6	0.864

For SA, we only found the existence of 2 participants whose pre-SA was over than the cut-off points, which did not reach the level of statistical analysis, so we only analysed the data within the cut-off points, and the results were the same as the

previous analysis. Specifically, the difference in pre-SA (t = 0.895 , df = 97, p = 0.373) and SCL (t = 0.357, df = 97, p = 0.772) between the competition and control groups was not significant, with the test scores competition group being better (t = -2.745, df = 97, p = 0.007), and the mediation of BIS in the mediation of SA on SCL on quizzes was significant (β = 0.0275, 95% CI = [0.05, 0.06]).

In study 2: For TA, we found that 6 participants had TA above the cut-off points, and further analyses revealed: 1) results of analyses within the cut-off point, similar to previous analyses. Specifically, the differences in TA and SCL between the competition and control groups were not significant, the test scores of the control group were slightly higher (Table S2), no mediate effect of BIS in TA on SCL. 2) Analyses of data outside the cut-off point show that none of the differences in TA, SCL, and test scores between the two groups, no mediate the effect.

Table S2 Results of cut-off points analysis of TA in Study 2

	within the cut-off point (N = 36)			without the cut-off point (N = 6)		
	t	df	p	t	df	p
TA	-1.505	34	0.142	-0.566	2	0.629
SCL	-1.074	32	0.291	-0.066	2	0.953
Test scores	1.827	33	0.077	-0.684	2	0.565

2) Analysis based on trait anxiety as a control variable

The results showed that the test scores and SCL results for both groups were consistent with the previous results. in Study 1, the test scores were higher in the competition group compared to the control group ($F_{(1, 97)}$ = 7.140, p = 0.009), and the

difference in the SCL between the two groups was not significant ($F_{(1, 97)} = 0.629$, $p = 0.430$); and in Study 2, the test scores were slightly lower in the competition group compared to the control group ($F_{(1, 32)} = 3.207$, $p = 0.063$), and the difference in SCL between the two groups was not significant ($F_{(1, 32)} = 0.619$, $p = 0.436$).

3) the results of five test scores of study 1

The repeated measures ANOVA results of five test scores showed that the main effect of time($F_{(4, 96)} = 134.577$, $p < 0.001$, $\eta^2 = 0.855$) and group($F_{(1, 99)} = 9.794$, $p = 0.012$, $\eta^2 = 0.063$) were significant, while the interaction between time and group was not significant. Competition group (8.70 ± 0.21) was higher than the control group (7.96 ± 0.20). The fifth quiz scored higher than that for other quizzes, the third and fourth quizzes were scored higher than the first and second quizzes, the first quiz was higher than the second quiz ($p < 0.05$).

4) **the results of five test scores of study 2**

The repeated measures ANOVA results of five test scores showed that the main effect of time($F_{(4, 34)} = 44.838$, $p < 0.001$, $\eta^2 = 0.841$) was significant and the main effect of group marginally significant($F_{(1, 37)} = 3.795$, $p = 0.059$, $\eta^2 = 0.093$), while the interaction between time were not significant. Competition group (8.66 ± 0.23) was lower than the control group (9.34 ± 0.27). The first and second quiz scored lower than the third, fourth, and fifth time, and the third and fourth scored lower than the fifth time ($p < 0.05$).
